# Serum muscle-derived enzymes response during show jumping competition in horse

**DOI:** 10.14202/vetworld.2016.251-255

**Published:** 2016-03-08

**Authors:** Anna Assenza, Simona Marafioti, Fulvio Congiu, Claudia Giannetto, Francesco Fazio, Daniele Bruschetta, Giuseppe Piccione

**Affiliations:** 1 Department of Cognitive Science, Education and Cultural Studies, University of Messina, Via Concezione n 6-8, 98122, Messina, Italy; 2 Department of Veterinary Sciences, University of Messina, Polo Universitario dell’Annunziata, 98168, Messina, Italy; 3 Department of Biomedical Sciences and Morpho-functional Imaging, University of Messina, Via Consolare Valeria 1, Messina, Italy

**Keywords:** horse, muscle enzymes, physical exercise, show jumping competition

## Abstract

**Aim:**

The effect of two jumping competitions, performed in two consecutive weekends, on serum creatine phosphokinase (CPK), aspartate aminotransferase (AST), and lactate dehydrogenase (LDH), urea, creatinine (CREA) concentrations were evaluated in 12 healthy jumper horses.

**Materials and Methods:**

Blood sampling was performed before the 1^st^ day of competition (T_0_), at the end of each show (J_1_, J_2_), on the day after the competition (T_1_); the same sampling plan was followed during the second weekend (J_3_, J_4_ and T_2_).

**Results:**

One-way repeated measures analysis of variance showed an increase in CPK at J_1_ and J_2_ respect to T_0_ and at J_3_ and J_4_ respect to all other time points (p<0.05). LDH activity showed an increase at J_2_ respect to T_0_, at J_3_ respect to T_0_, J_1_, J_2_ and at J_4_ respect to all other time points (p<0.05). AST values increased at J_1_ and J_2_ respect to T_0_ (p<0.05). A significant increase of CREA was found at J_3_ respect to T_0_, T_1_ and J_1_ and at J_4_ respect to all other time points (p<0.05). A decrease in serum urea levels was found at J_1_ respect to T_0_, at J_2_ and J_4_ respect to T_0_ and T_1_; at T_2_ respect to T_0_ (p<0.05). A positive correlation between urea/CPK (p=0.0042, r^2^=0.030), LDH/CPK (p<0.0001, r^2^=0.535), CREA/LDH (p<0.0001, r^2^=0.263), CREA/CPK (p<0.0001, r^2^=0.496) was observed.

**Conclusion:**

Our results suggest that 5 days recovery period between the two consecutive competition weekends is insufficient to allow muscle recovery and avoid potential additional stress. The findings obtained in this study improve the knowledge about metabolic changes occurring in athlete horse during the competition to identify muscle alterations following show jumping competitions.

## Introduction

The performance of athletic horse is determined by many complicated interdependent biological and physiological processes. Similarity to other stressors, including delivery, transport and environmental conditions exercise need adequate response to re-establish homeostatic equilibrium [1]. Several cardiovascular and hematological adaptations are necessary to guarantee the correct supply of oxygen to active muscles during exercise. Physiological, hematological, and biochemical changes associated with exercise have been extensively analyzed in several types of horses such as thoroughbreds [2,3] eventers [4,5], show jumpers [6-9], and endurance horses [10,11].

One of the organs affected by exercise is the muscle, which suffers microdamage due to effort employed load [12]. Many researchers have compared muscular adaptations that occur after several training programs with different exercise intensities [13,14], others have examined the combined effect of intensity and duration of the exercise [15], or assessed adequate recovery after exercise.

Repeated workload leads the muscular apparatus to constant damage that can be assessed by laboratorial determination of some serum constituent such as urea and creatinine (CREA) and enzymes such as creatine phosphokinase (CPK), aspartate aminotransferase (AST), and lactate dehydrogenase (LDH) [16]. The activity of these enzymes has been studied by several researchers before and after exercise and can be used to detect muscle diseases [17], characterization of exercise intensity [11,18] and predicting possible complications that can arise from the exercise [19]. Despite the existing literature describing muscular adaptations to training in horses, little is still known about the durations and intensities of exercise that promote optimal response in skeletal muscles [20]. Considering the metabolic and clinical role of serum parameters above mentioned and considering the effect of physical exercise on them, studying changes in these muscle damage markers after exercise has become more important [21].

Therefore, the objective of this study was to evaluate the serum concentration of urea, CREA and the serum activities of CPK, AST and LDH of jumper horses before and after jumping competition.

## Materials and Methods

### Ethical approval

Protocols of animal husbandry and experimentation were reviewed and approved in accordance with the standards recommended by the Guide for the Care and Use of Laboratory Animals and Directive 2010/63/EU for animal experiments.

### Animals

The study was carried out on 12 healthy and regularly trained Italian Saddle horses (7 geldings and 5 females, 9-12-year-old, mean body weight 500±20 kg). All horses were managed equally, housed in individual boxes under natural photoperiod (mean temperature 25±6°C, relative humidity 67±3%). The horses were fed standard rations, calculated to fulfill all the nutritional requirements according to National Institute of Agronomic Research specifications [22] constituted hay (first cut meadow hay, sun cured, late cut, and a mixture of cereals) oats and barley, 50% each.

The percentage composition of the mixture was dry matter 87% and moisture 13%. The dry matter contained 9.11% digestible protein, 13.05% crude protein, 20.7% crude fiber, and 3.42% crude lipid, as well as 0.80 Unitè Fouragire Cheval/kg. The ration was administered 3 times a day: 8:00 AM, 12:00 PM and 5:00 PM. Water was available *ad libitum*.

### Show jumping course

Horses took part in jumping competitions held in two consecutive courses at distance of 5 days. Each completion session was preceded by 20 min warm-up consisting of walk, trot and gallop with six jumps (height: From 100 to 140 cm). During the 1^st^ day of both weekends, horses competed with the following technical specifications: Total length - 550 m; obstacles height - 140 cm; total efforts 13 (7 verticals, 6 oxers, 1 triple combination). During the 2^nd^ day of both weekend competitions, horses competed with the following technical specifications: Total length - 600 m; obstacles height - 145 cm; and mixed competition including efforts 15 (8 verticals, 7 oxers, 1 double combination, 1 triple combination).

All horses during the week between both competitions performed the following daily training schedule warm-up (10 min walk, 20 min trot, 10 min gallop) and show jumping course with 7 fences of 80±10 cm average height.

### Blood sampling and analysis

Blood samples were collected by jugular venipuncture in vacutainer tubes with cloth activator for serum analyses (Terumo Co., Tokyo, Japan). Blood sampling was performed before the 1^st^ day of competition (T_0_), within 10 min from the end of each competition (J_1_, J_2_) and on the day after competition (T_1_), same plan was followed during second weekend (J_3_, J_4_, and T_2_). Immediately after collection, blood samples were placed in refrigerated bags and transported to the laboratory for the analysis. Tubes were centrifuged at 3000 rpm for 10 min and on obtained sera the concentration of CPK, LDH, AST, urea, CREA were determined by commercial available kit by means of an automated analyzer, Model 7070 (Hitachi Ltd., Tokyo).

The same operator assayed all samples in duplicate each time. Samples exhibited parallel displacement to the standard curve; the intra-assay and the inter-assay coefficients of variation were <7% and <9%, respectively, for all measured parameters.

### Statistical analysis

The obtained data are expressed as mean ± standard deviation (SD) of the mean. Data were normally distributed (p>0.05, Kolmogorov–Smirnov test). One-way repeated measures analysis of variance (ANOVA) was applied to determine the statistically significant effect of exercise on all parameters. p<0.05 were considered statistically significant. Bonferroni’s multiple comparison tests were applied for *post-hoc* comparison. A simple linear regression model was applied to evaluate the correlation between the studied parameters. A statistical analysis was performed using Stats package of R Core Team (2013) (R: A language and environment for statistical computing. R Foundation for Statistical Computing, Vienna, Austria. ISBN 3-900051-07-0, 2013, URL: http://www.R-project.org/).

## Results

ANOVA showed an increase in CPK at J_1_ and J_2_ respect to T_0_ and at J_3_ and J_4_ respect to all other time points. LDH activity showed an increase at J_2_ respect to T_0_, at J_3_ respect to T_0_, J_1_, J_2_ and at J_4_ respect to all other time points. AST values increased at J_1_ and J_2_ respect to T_0_. A significant increase of CREA was found at J_3_ respect to T_0_, T_1_ and J_1_ and at J_4_ respect to all other time points. A decrease in serum urea levels was found at J_1_ respect to T_0_, at J_2_ and J_4_ respect to T_0_ and T_1_; at T_2_ respect to T_0_.

**Figure-1:**
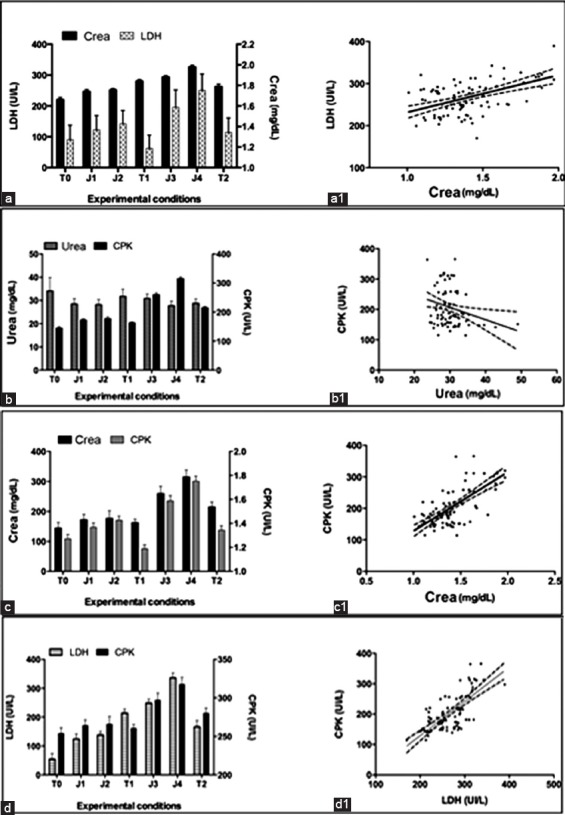
(a) Pattern of serum creatinine (CREA) and lactic dehydrogenase (LDH) observed during experimental period; (a1) Graphical representation of simple linear regression of LDH vs. CREA; (b) Pattern of CREA and creatine phosphokinase (CPK) observed during experimental period; (b1) Graphical representation of simple linear regression of CPK versus CREA, (c) Pattern of serum urea and CPK observed during experimental period; (c1) Graphical representation of simple linear regression of CPK versus urea; (d) Pattern of LDH and CPK observed during experimental period; (d1) graphical representation of simple linear regression of CPK versus LDH.

A positive correlation between CREA/LDH (p<0.0001, r^2^=0.263), CREA/CPK (p<0.0001, r^2^=0.496) urea/CPK (p=0.0042, r^2^=0.030), LDH/CPK (p<0.0001, r^2^=0.535), was observed (Figure-1).

## Discussion

The obtained results showed that hematochemical modifications occur after exercise in jumper horses. With regard to the muscle enzymes, an increase in the post-exercise activities of CPK, AST and LDH was fond compared to baseline values measured at T_0_. According to our results, increase in serum CPK, AST, and LDH activities have been seen in response to exercise [23]. These increases are believed to relate either to overt damage or to a change in the muscle fiber membrane causing a transient increase in permeability [24]. However, physiological increases have been also shown to occur without any tissue alteration [25]. The effects of physical effort on serum enzymatic activity may depend on the level of performance of the animal, and the intensity and duration of exercise [26].

A significant increase of CPK was observed after the competitions. The post-exercise increase of CPK levels could be attributed to the muscle metabolism and to the increasing energy requirements occurring during physical exercise [27]. It is well stated that the tissue CPK activity may augment energetic capacity and improve myofibril contraction responses through enhancing vascular tone and vasoconstrictor reserves [27]. At T_1_, it showed slightly, but no significant decrease respect to J_1_ and J_2_, although remained highest, but no significantly, respect to T_0_. This probably because blood sampling at T_1_ occurred 24 h after the first weekend of competition and this is an insufficient time to determine a return to normal serum concentration. During the second weekend of competition, CPK assumed higher levels respect to the previous week and reached its peak levels in J_4_. Its values remained significantly higher at T_2_ respect to T_0_ assuming that the shorter recovery period to the first competition (only 4 days) associated with another rise post-exercise, could increase the probability of muscle damage. CPK has relatively shorter half-life of 2 h [28]. Therefore, will become elevated sooner and return to normal range after an episode of muscle strain, unless the effort is so high as to delay its return to the baseline levels. For this reason, it is preferred to evaluate muscle enzyme for diagnosing and monitoring muscle recovery and considered a reliable marker of skeletal muscle injury [28]. LDH activity showed a significant increase at J_2_ respect to T_0_, probably due both to the increased effort respect to J_1_ and to the increase of enzyme activity after 24 h post exercise (T_1_). LDH have a longer half-life respect to CPK, in fact, it peaks 24 h after effort and could remain high for 48 h after exercise [11]. Therefore, since blood sampling at T_1_ occurred 24 h after the last jumping round of the 1st week of completion, return to baseline levels could not be determined. Our study showed a greater increase in the levels of this enzyme at J_3_ and J_4_ revealing, as well as in CPK activity, a reduced capacity to recovery by effort probably due to the horses have performed competitions in two consecutive weekend.

AST activities may increase during exercise without observation of clinical signs or histological detection of changes in muscle cell structure [29]. In our study, AST values increased immediately after exercise, whereas they decreased at T_1_ reaching levels measured at T_0_.

In this study, a slightly but significant increase of CREA was found in J_3_ and J_4_. CREA is produced from the decomposition of creatine, a nitrogen compound used by muscle cells to store energy. The serum concentration of CREA varies according to creatine synthesis and the amount of muscle mass and exercise as reported by Nogueira *et al*. [30] in thoroughbred.

During the exercise phases and the recovery period, all animals showed a decrease in serum Urea levels respect to T_0_. Urea is filtered by the glomerular capillaries, and it enters the renal tubule. Approximately half of urea is reabsorbed passively by diffusion, but the remainder is excreted in the urine. Lower urea levels suggest an increased glomerular filtration and an excretion in the urine or a diminished reabsorption in the tubules.

On the basis of our results, we can affirm that the higher levels of CPK and LDH in J_2_ and J_4_ (increased effort) respect to J_1_ and J_3_ confirmed that the effects of physical effort on serum activities of muscle enzymes is strictly linked with intensity and duration of exercise; the increased levels of CPK and LDH occurred during the second weekend of competition (J_3_ and J_4_) respect to the first, seem to indicate that the two jumping session were temporally too close and did not allow the horse adequate recovery.

## Conclusion

Our results improved the knowledge about metabolic changes occurring in athlete horse during competition and underline that the physiological activity of enzymes, if not associated with the adequate recovery period, can increase the probability of muscle damage.

## Authors’ Contributions

AA and GP designed the study and supervised the research as major advisor. SM, FC and FF worked and collaborated in the lab work and compilation of the results as well as the manuscript. GC and DB provided valuable suggestions regarding the design of the experiment and analysis of the data collected during research. All authors read and approved the final manuscript.

## References

[1] Piccione G, Rizzo M, Arfuso F, Giannetto C, Di Pietro S, Bazzano M, Quartuccio M (2015). Leukocyte modifications during the first month after foaling in mares and their newborn foals. Polish J. Vet. Sci.

[2] Fazio F, Assenza A, Tosto F, Casella S, Piccione G, Caola G (2011). Training and haematochemical profile in thoroughbreds and standard breds: A longitudinal study. Livest. Sci.

[3] Mukai K, Takahashi T, Eto D, Ohmura H, Tsubone H, Hiraga A (2007). Heart rates and blood lactate response in thoroughbreds horses during a race. J. Equine Sci.

[4] Muñoz A, Riber C, Santisteban R, Rubio M.D, Agüera E.I, Casteión F.M (1999). Cardiovascular and metabolic adaptations in horses competing in cross-country events. J. Vet. Med. Sci.

[5] White S.L (1998). Fluid, electrolyte, and acid-base balances in three-day, combined-training horses. Vet. Clin. North Am. Equine Pract.

[6] Assenza A, Bergero D, Congiu F, Tosto F, Giannetto C, Piccione G (2014). Evaluation of serum electrolytes and blood lactate concentration during repeated maximal exercise in horse. J. Equine Vet. Sci.

[7] Piccione G, Giannetto C, Assenza A, Fazio F, Caola G (2007). Serum electrolyte and protein modification during different workload in jumper horse. Comp. Clin. Path.

[8] Piccione G, Casella S, Giannetto C, Messina V, Niutta P.P, Giudice E (2011). Effect of hydrocortisone on platelet aggregation in jumper horses. Vet. Arch.

[9] Fazio F, Casella S, Assenza A, Arfuso F, Tosto F, Piccione G (2014). Blood biochemical changes in show jumpers during a simulated show jumping test. Vet. Arch.

[10] Muñoz A, Cuesta I, Riber C, Gata J, Trigo P, Castejon F.M (2006). Trot asymmetry in relation to physical performance and metabolism in equine endurance rides. Equine Vet. J.

[11] Teixeira-Neto A.R, Ferraz G.C, Moscardini A.R.C, Balsamão G.M, Souza J.C.F, Queiroz-Neto A (2008). Alterations in muscular enzymes of horses competing long-distance endurance rides under tropical climate. Arq. Bras. Med. Vet. Zootec.

[12] Brioschi Soares O.A, de Freitas D’angelis F.H, Feringer W.H, Bacciotti Nard I.K, Trigo P, Queiroz de Almeida F, Tavares Miranda A.C, Queiroz-Neto A, de Camargo Ferraz G (2013). Serum activity of creatine kinase and aminotransferase aspartate enzymes of horses submitted to muscle biopsy and incremental jump test. Rev. Bras. Saúde Prod. Anim.

[13] Eaton M.D, Hodgson D.R, Evans D.L, Rose R.J (1999). Effects of low- and moderate-intensity training on metabolic responses to exercise in thoroughbreds. Equine Vet. J. Suppl.

[14] Sinha A.K, Ray S.P, Rose R.J (1993). Effect of constant load training on skeletal muscle histochemistry of thoroughbred horses. Res. Vet. Sci.

[15] Gansen S, Lindner A, Marx S, Mosen H, Sallmann H.P (1999). Effects of conditioning horses with lactate-guided exercise on muscle glycogen content. Equine Vet. J. Suppl.

[16] Overgaard K, Fredsted A, Hyldal A, Gissel H, Clausen T (2004). Effects of running distance and training on Ca^2+^ content and damage in human muscle. Med. Sci. Sports Exerc.

[17] Boffi F.M (2007). Pathologies affecting the athletic performance. Muscle disorders. Equine Exercise Physiology.

[18] Ferraz G.C, Soares O.A.B, Foz N.S.B, Pereira M.C, Queiroz-Neto A (2010). The workload and plasma ion concentration in a training match session of high-goal (elite) polo ponies. Equine Vet. J.

[19] Trigo P, Castejon F, Riber C, Muñoz A (2010). Use of biochemical parameters to predict metabolic elimination in endurance rides. Equine Vet. J. Suppl.

[20] Rivero J.L.L, Ruz A, Martí-Korff S, Estepa J.C, Aguilera-Tejero E, Werkman J, Sobotta M, Lindner A (2007). Effects of intensity and duration of exercise on muscular responses to training of thoroughbred racehorses. J. Appl. Physiol.

[21] Amir-Shaqaqi M (2006). Effects of a Period of Selected Plyometric Exercises on LDH, CK, and UREA Levels in Elite Female Soccer Players.

[22] Martin-Rosset W (1990). Horse nutrition. INRA Editions.

[23] Harris P.A, Reed S.M, Bayly W.M (1998). Musculoskeletal disease. Equine Internal Medicine.

[24] Anderson M.G (1975). The influence of exercise on serum enzyme levels in the horse. Equine Vet. J.

[25] Valberg S, Johnson L, Lindholm A, Holmgren N (1993). Muscle histopathology and plasma aspartate aminotransferase, creatine kinase and myoglobin changes with exercise in horses with recurrent exertional rhabdomyolysis. Equine Vet. J.

[26] Bogdanis G.C (2012). Effects of physical activity and inactivity on muscle fatigue. Front. Physiol.

[27] Brewster L.M, Mairuhu G, Bindraban N.R, Koopmans R.P, Clark J.F, Van Montfrans G.A (2006). Creatine kinase activity is associated with blood pressure. Circulation.

[28] Octura J.E.R, Lee K.J, Cho H.W, Vega R.S.A, Choi J.Y, Park J.W, Shin T.S, Cho S.K, Kim B.W, Cho B.W (2014). Elevation of blood creatine kinase and selected blood parameters after exercise in thoroughbred racehorses (*Equus caballus* L.). J. Res. Agric. Anim. Sci.

[29] Câmara E, Silva I.A, Dias R.V.C, Soto-Blanco B (2007). Determination of serum activities of creatine kinase, lactate dehydrogenase, and aspartate aminotransferase in horses of different activities classes. Arq. Bras. Med. Vet. Zootec.

[30] Nogueira G.P, Barnabe R.C, Bedran-De-Castro J.C, Moreira A.F, Fernandes W.R, Mirandola R.M.S, Howard D.L (2002). Serum cortisol, lactate and creatinine concentrations in Thoroughbred fillies of different ages and states of training. Braz. J. Vet. Res. Anim. Sci.

